# Temporal variations of black carbon during haze and non-haze days in Beijing

**DOI:** 10.1038/srep33331

**Published:** 2016-09-16

**Authors:** Qingyang Liu, Tangming Ma, Michael R Olson, Yanju Liu, Tingting Zhang, Yu Wu, James J. Schauer

**Affiliations:** 1 College of Biology and the Environment, Collaborative Innovation Center of Sustainable Forestry in Southern China of Jiangsu Province, Nanjing Forestry University, Nanjing, China; 2 Environmental Chemistry and Technology Program, University of Wisconsin-Madison, Madison, WI, USA; 3 Beijing Center for Physical and Chemical Analysis, Beijing, China; 4 Wisconsin State Laboratory of Hygiene, University of Wisconsin-Madison, Madison, WI, USA

## Abstract

Black carbon (BC) aerosol has been identified as one of key factors responsible for air quality in Beijing. BC emissions abatement could help slow regional climate change while providing benefits for public health. In order to quantify its variations and contribution to air pollution, we systematically studied real-time measurements of equivalent black carbon (eBC) in PM_2.5_ aerosols at an urban site in Beijing from 2010 to 2014. Equivalent black carbon (eBC) is used instead of black carbon (BC) for data derived from Aethalometer-31 measurement. Equivalent BC concentrations showed significant temporal variations with seasonal mean concentration varying between 2.13 and 5.97 μg m^−3^. The highest concentrations of eBC were found during autumn and winter, and the lowest concentrations occurred in spring. We assessed the temporal variations of eBC concentration during haze days versus non-haze days and found significantly lower eBC fractions in PM_2.5_ on haze days compared to those on non-haze days. Finally, we observed a clear inverse relationship between eBC and wind speed. Our results show that wind disperses PM_2.5_ more efficiently than eBC; so, secondary aerosols are not formed to the same degree as primary aerosols over the same transport distance during windy conditions.

Black carbon (BC) aerosol is formed by the incomplete combustion of fuels and is an important atmospheric components because of its potentially negative effects on climate and health^1^,^2^,^3^. BC absorbs visible solar radiation in the atmosphere, and has been identified as a major contributor to global warming^4^,^5^,^6^. The direct effects of BC are different from the warming effect of greenhouse gases, for BC causes atmospheric heating and surface cooling, while greenhouse gases heat both the atmosphere and the Earth’s surface^5^,^6^. Intense atmospheric heating caused by BC is regarded as an important contributor to the retreat of Himalayan glaciers^7^,^8^. Not only is BC a climate forcing agent, it is also associated with many respiratory diseases and detrimentally affects the cardiovascular system^9^,^10^,^11^,^12^,^13^.

A technology-based global BC emission inventory estimated annual emissions of BC from 50 anthropogenic sources to be 4669 Gg in 1996; combustion of fossil fuels, solid fuels, and open burning comprised approximately 38%, 20%, and 42% of this estimate, respectively^14^. Previous results indicate that BC sources including industrial, transportation, and residential sources vary significantly among countries, especially between developing and developed countries^15^,^16^,^17^. The main source of BC in the developed continents of North America and Europe is transportation^15^, whereas the combustion of fossil fuel dominates in the developing continents such as Africa and most of Asia^14^. The emissions of BC and other pollutants from Asian regions could significantly influence the air quality in downwind regions, especially during the East Asia northwesterly monsoon season^14^. China is the largest emitter of BC in Asia, and the predominant sources include residential combustion (55.3%), industrial emissions (31.8%), transportation (10.9%) and power production (1.8%)^14^.The transportation sector has been recognized as a significant contributor to BC emissions in the urban atmosphere of China^18^. Although BC emissions from transportation are now two times lower in China than in India, a previous study predicted that BC emissions from transportation in China would surpass those in India in 2020, and China would subsequently become the largest BC emitter in Asia^19^. Since previous research has been unable to accurately predict urban BC sourced from transportation, long-term measurement of these emissions is necessary in order to develop an appropriate mitigation strategy for BC in China.

Numerous Chinese cities have suffered from serious air pollution in recent years, and it has become one of the nation’s top environmental concerns^20^,^21^,^22^,^23^,^24^. As a result of rapid energy consumption and urbanization, there are high concentrations of PM_2.5_ in Beijing, which causes frequent haze episodes in the area^20^,^21^. During January 2013, 80% of the days in the month were categorized as haze days. The highest daily concentration was 755 μg m^−3^, which is over 30 times greater than the World Health Organization (WHO) daily Air Quality Guideline (25 μg m^−3^)^21^. Previous field studies have suggested that the main factors for haze formation in Beijing are meteorological conditions^22^ and secondary aerosols (i.e., secondary organic aerosol and secondary inorganic aerosol)^20^,^21^,^24^. Primary emissions including vehicles (6–9%), industry (5–10%), biomass burning (5–7%) and residential coal burning (3–26%) also play a small role in haze formation^20^,^21^,^24^. BC is an important component of PM_2.5_ and is an indicator of primary emissions. Daily BC concentrations have strong day-to-day variations, and an average BC concentration during a severe haze episode in January 2013 (7.6 ± 4.8 μg m^−3^) is 2.5 times greater than on clear days (2.0 ± 1.2 μg m^−3^)^24^. BC aerosol is also a major substrate for secondary aerosol transformation due to its porous and adsorptive nature. Recent studies indicate that BC aerosol in Beijing is more easily transformed from a fractal to spherical morphology than those in Houston (2.3 hours versus 9 hours); this transformation is an essential indicative for secondary aerosol formation in the urban atmosphere^25^. Therefore, it is plausible that BC aerosols in polluted urban environments share common properties with BC particles in developed countries. Fully understanding the large variability of BC in urban polluted atmospheres could bolster policies’ effectiveness in mitigating haze pollution in developing countries like China, where a large population faces considerable health risks associated with exposure to air pollution.

For 5 years, equivalent BC was continuously measured at a roadside site in urban Beijing and analyzed for daily, seasonal, and annual variations in order to identify how they relate to source emissions and meteorology. We focused on primary emissions by using equivalent BC as a representative aerosol, and we addressed the following points: (1) the sources and meteorological factors responsible for variations in equivalent BC, and (2) changes in equivalent BC concentrations and equivalent BC fractions in PM_2.5_ during haze episodes. This study contributes to field of emission abatement by providing new information regarding measurement of equivalent BC concentrations and identification of sources. It is complemented by parallel studies concerning optimization of air quality regulations for developing countries, such as China.

## Results and Discussion

### Daily equivalent BC and PM_2.5_ mass concentration

**Figure 1:**
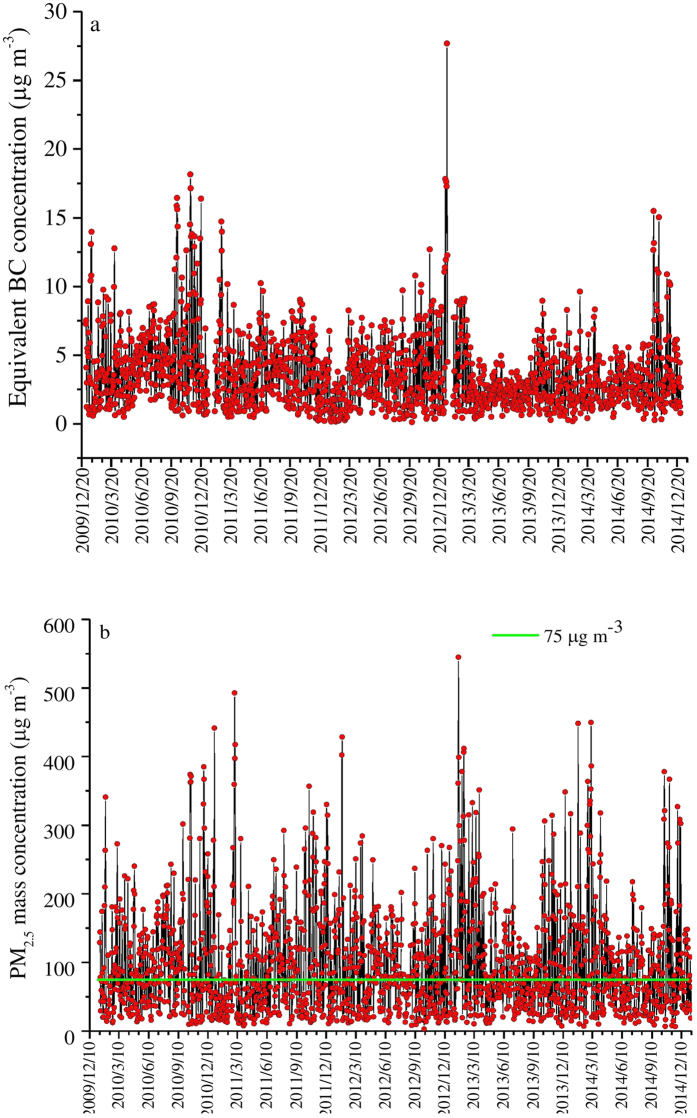
The time series of daily equivalent BC (**a**) and PM_2.5_(**b**) mass concentration at a roadside in Beijing from 2010 to 2014. 75 μg m^−3^ is the Chinese standard of PM_2.5_ pollution. Missing data is due to instrument failure.

Figure 1a shows the daily-mean temporal variations of equivalent BC from January 1, 2010 to December 30, 2014. A total of 1756 daily equivalent BC samples were collected with mass concentrations ranging from 0.16 to 27.69 μg m^−3^, and the average is 3.67 μg m^−3^. High daily mean equivalent BC concentrations (>10 μg m^−3^) were mostly observed during the winter months (December-February), and the lowest concentrations were observed during the spring months (March-May). The observed mean BC concentration in this study is much lower than those in megacities of India, such as Agra (20.6 μg m^−3^) and Mumbai (12.5 μg m^−3^), as well as those in Beijing during the years of 1999–2000 (8.7 μg m^−3^), 2001–2003 (20.9 μg m^−3^), and 2005–2006 (6.5 μg m^−3^)^26^, indicating that BC concentration in Beijing has been steadily decreasing.

The daily average PM_2.5_ mass concentration varied from 3 to 545 μg m^−3^, with a mean value of 97 μg m^−3^ (Fig. 1b), which exceeds the upper limit value of PM_2.5_ mass (75 μg m^−3^) for air pollution in China. Over the 5-year measurement period, 53.6% of PM_2.5_ mass concentrations exceeded this upper limit value. This high percentage of PM_2.5_ concentration over the standard limit for air pollution (75 μg m^−3^) indicates that strict controls over PM sources in Beijing are required^24^.

The average ratio for equivalent BC/PM_2.5_ ratio is 4.6%, which falls within the range of 0.2% to 26.9% for individual PM_2.5_ samples (Table S1). The eBC/PM_2.5_ is mainly affected by the burning of different fossil fuels^26^,^27^,^28^. The source apportionment results found that traffic-related emissions were the dominant BC source in Beijing throughout the whole year, with a mean contribution of 79 ± 6%^29^. It was also found that coal combustion made a greater contribution to BC concentration in the cold season (19%) than in the warm season (3%)^29^. The large variations in equivalent BC/PM_2.5_ ratios could be attributed to the differences in emission contributions between seasons.

The BC and PM_2.5_ sampling sites, which are located 1.4 km apart, share a proximal orientation to the west 3^rd^ highways that pass through this region of Beijing (Figure S1). Both sites are downwind receptors of emissions from the west 3^rd^ highways and are impacted by them equally from all wind directions^30^,^31^. Many studies have observed that PM_2.5_ composition in Beijing is strongly affected by the distance between the air sampling location and the emission sources^29^,^32^,^33^. Previous source apportionment results have found that traffic emissions are the primary source of BC aerosol in urban Beijing, whereas biomass BC aerosol plays the predominant role in the suburbs^32^,^33^ Since both the sampling sites are oriented similarly to the 3^rd^ highway and are similarly impacted by the roadway at all wind directions, the uncertainty due to variability in site location is insignificant.

### Seasonal equivalent BC and PM_2.5_ mass concentration

**Figure 2:**
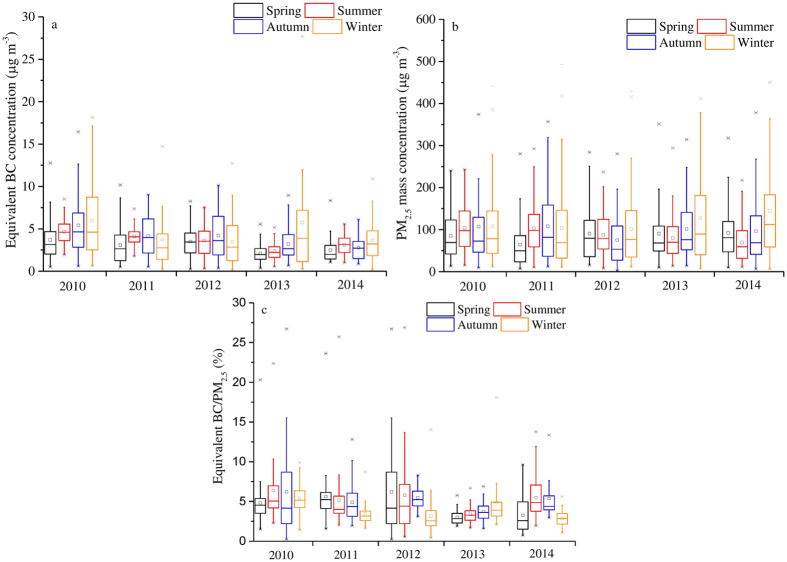
Seasonal trends of equivalent BC concentration (**a**), PM_2.5_ mass concentration (**b**) and equivalent BC/PM_2.5_ (**c**) of Beijing from 2010 to 2014. All data points are shown in the box and whisker plots: the band near the middle of the box is the median value of the data; the dot in the box is the arithmetic mean value of the data; the top end of the box is 25% of the data and the lower end the box is 75% of the data; whiskers indicate distribution of minimum and maximum values.

The seasonal summaries of equivalent BC and PM_2.5_ mass values for each year are shown in Fig. 2 and Table S1. The individual lowest average for each year occurred in the spring (3.71 μg m^−3^ in 2010, 3.09 μg m^−3^ in 2011, 3.50 μg m^−3^ in 2012, 2.13 μg m^−3^ in 2013, and 2.47 μg m^−3^ in 2014); the second lowest occurred during summertime (4.66 μg m^−3^ in 2010, 4.10 μg m^−3^ in 2011, 3.63 μg m^−3^ in 2012, 2.36 μg m^−3^ in 2013, and 3.11 μg m^−3^ in 2014); and the highest occurred in autumn (4.16 μg m^−3^ in 2011 and 4.21 μg m^−3^ in 2012) and winter (5.97 μg m^−3^ in 2010, 5.74 μg m^−3^ in 2013, and 3.62 μg m^−3^ in 2014). Our results support a previous study that found that residential coal burning during periods with low temperatures and relatively stable meteorological conditions could result in increases in BC concentration during the cold season^22^,^29^. However, this previous study also states that PM_2.5_ concentrations and equivalent BC fractions in PM_2.5_ concentrations exhibit no obvious seasonal variations. This is inconsistent with the results of the current study, since it was found that PM_2.5_ concentrations were considerably higher during the wintertime of the years 2010–2014. A larger contribution from coal combustion during the winter season in Beijing may be the cause of the dramatic increase in PM_2.5_ concentrations^24^,^34^ Accurate information on the highly varying emission factors of BC combustion sources is needed to illustrate the inter-seasonal differences, which are all based on emission inventories^35^. However, detailed emission inventories in developing countries (e.g., China) have not been fully understood yet^14^,^35^. Incomplete understanding of BC emission inventories is the cause for uncertainty in explaining the inter-seasonal differences in this study; thus, it will be independently evaluated in a further study.

### Annual equivalent BC and PM_2.5_ mass concentration

**Figure 3:**
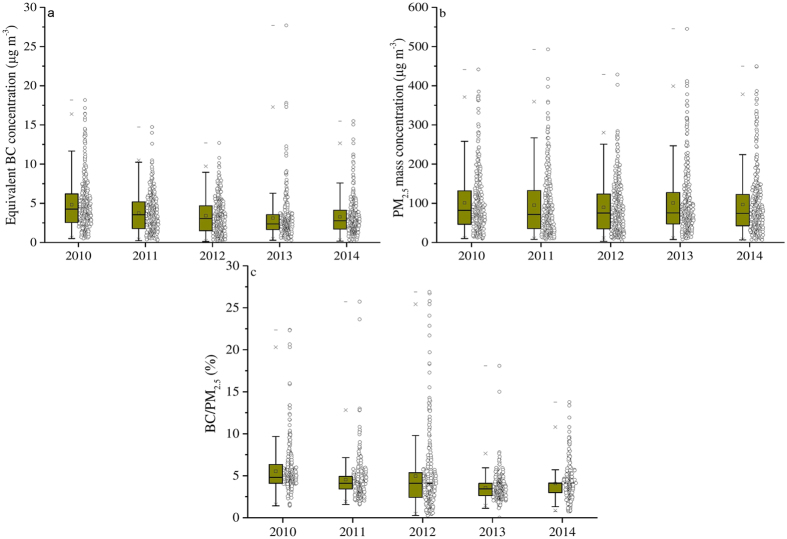
Annual trends in equivalent BC concentration (**a**), PM_2.5_ mass concentration (**b**) and equivalent BC/PM_2.5_ (**c**) of Beijing in 2010 to 2014. All data points are shown in the box and whisker plots: the band near the middle of the box is the median value of the data; the dot in the box is the arithmetic mean value of the data; the top end of the box is 25% of the data and the lower end the box is 75% of the data; whiskers indicate distribution of minimum and maximum values.

Annual mean equivalent BC and PM_2.5_ concentrations are shown in Fig. 3 and Table S2. The annual mean equivalent BC concentration was 4.82 μg m^−3^ in 2010, and the 2011 annual mean equivalent BC concentration of 3.80 μg m^−3^ is 20% lower than that of 2010 (significant at 95% CI, p < 0.05). Concentrations remained relatively uniform within 9% between 2011 and 2012, with a slightly higher variation of 20% from 2012 to 2013. However, equivalent BC concentration was 3.27 μg m^−3^ in 2014—11% higher than that of 2013 (2.95 μg m^−3^) (Table S2). Mean PM_2.5_ mass concentrations show a downward trend from 2010 to 2013, within a 10% variation. PM_2.5_ mass concentration steadily increased throughout the period increasing by 11 μg m^−3^ (10%) from 2012 to 2013., and it remained relatively uniform within 2% between 2013 and 2014 (Fig. 3). The annual mean equivalent BC/PM_2.5_ ratio was 5.7% in 2010, which is approximately 10% higher than the ratios in 2011 (4.7%) and 2012 (5.0%). Thereafter, equivalent BC fractions decreased to 3.6% in 2013, with a slight increase to 4.3% in 2014; overall, there was a 1.4% decrease in the equivalent BC/PM_2.5_ ratio since 2010 (5.7%).

Wang *et al.*^14^ calculated the annual BC emissions from various sources in China from 1949 to 2020 and found that the largest source for BC was residential coal combustion, followed by residential biomass burning, diesel vehicles and coke production. The emissions from the residential sector and coke production have declined since 1990 in urban Beijing because coal stoves have been replaced with liquid petroleum gas stoves and centralized heating systems has expanded^14^,^32^. Furthermore, the implementation of new emission regulations for gasoline and diesel vehicles (National Stage IV for emission regulation) starting in 2004 in Beijing is expected to reduce BC emissions^36^. Outdoor biomass burning accounts for a relatively small fraction of BC emissions (~15%) in Beijing and has had limited influence on the temporal trend of Central Beijing, since very high emission densities of biomass burning can only be found in rural area of Beijing^14^,^24^,^29^. Considering the number of on-road diesel vehicles has remained steady over the past five years (Table S3)^36^, the slight decrease in Beijing’s BC concentrations can be attributed to advances in technology and compliance to control facilities^37^,^38^.

### Characteristics of equivalent BC aerosols during haze days and non-haze days

**Figure 4:**
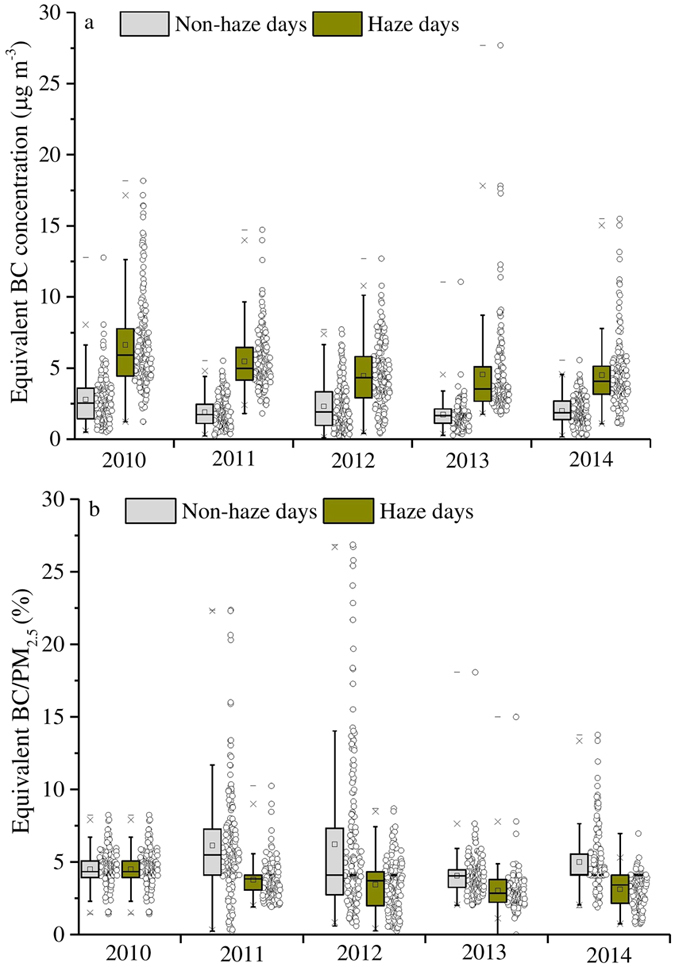
Data summary of equivalent BC concentration (**a**) and equivalent BC/PM_2.5_ (**b**) in Beijing on haze and non-haze days from 2010 to 2014. All data points are shown in the box and whisker plots: the band near the middle of the box is the median value of the data; the dot in the box is the arithmetic mean value of the data; the top end of the box is 25% of the data and the lower end the box is 75% of the data; whiskers indicate distribution of minimum and maximum values.

As shown in Fig. 4 and Table S4, the average concentrations of equivalent BC on haze days were 1–2 times higher than those on non-haze days. The significant difference in equivalent BC concentrations between haze days versus non-haze days is probably due to greater direct primary emissions and relatively stagnant meteorological conditions during haze days. These findings are consistent with numerous previous studies that found that air pollutants reach their peak concentration during haze episodes^29^,^22^,^24^. The ratios of equivalent BC and PM_2.5_ were considerably lower (about 1–2 times on average) on haze days than on normal days, which indicates that BC emissions are not the main factor contributing to Beijing haze formation during the five studied years. Wu *et al.*^39^ also observed a decreased equivalent BC fraction in PM_2.5_ during the haze episode of January, 2013.

### Relationships between equivalent BC concentration and meteorological factors

**Figure 5:**
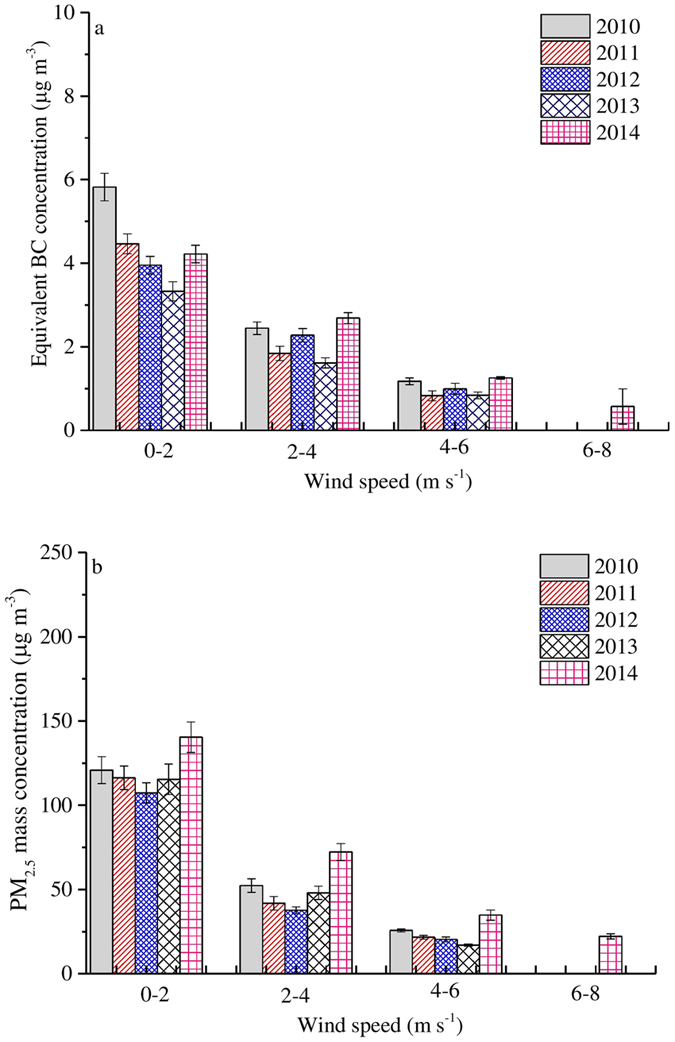
Equivalent BC (**a**) and PM_2.5_ mass concentrations (**b**) as function of wind speed from 2010 to 2014. The error bars denote standard error.

Daily meteorological measurements including temperature, relative humidity (RH), pressure, wind speed, wind direction, and rainfall were collected and are provided in the Supplementary Information (Table S5). The daily mean temperature, RH, pressure, wind speed and rainfall were 12 ^o^C, 54%, 1021 hPa, 1.8 m s^−1^ and 565 mm, respectively. The relationship between meteorological parameters, equivalent BC, and PM_2.5_ has been examined (Table 1), and it was found that both equivalent BC and PM_2.5_ concentration are significantly negatively correlated with wind speed (r = 0.46–0.47, p < 0.001). This is consistent with previous results that locally generated BC could accumulate under low wind speed conditions^40^. Jones *et al.*^41^ found that that increased wind speeds result in a general reduction of airborne particulate concentrations due to increased vertical dispersion. More rapid reductions in particulate concentrations occur within the vicinity of discrete point or line sources at high wind speeds due to dilution at the source^41^. Plots of equivalent BC versus wind speed (Figure S2a) and PM_2.5_ concentration versus wind speed (Figure S2b) show that concentrations decrease with higher wind speeds (6–8 m s^−1^), which is evidence of dilution. Figure 5a shows a higher dilution rate than Fig. 5b at the same wind speed (50% versus 35%, respectively), indicating that wind disperses PM_2.5_ more efficiently than equivalent BC (Fig. 5). Secondary aerosol formation is much quicker during stagnant versus windy conditions. This is supported by well-documented observations at urban sites in Beijing^20^,^21^ and Shanghai^21^,^42^. A possible explanation is that secondary aerosols easily formed to the same degree over the same transport distance under stable conditions compared to those under windy conditions^20^. Under the stable conditions and polluted atmosphere, the oxidation rates of secondary aerosol precursors with OH radical are considerably higher those in windy conditions, resulting in greater amounts of secondary aerosols^21^,^42^. Additionally, secondary aerosols may contribute more to PM_2.5_ mass concentration than primary aerosols in Beijing^21^,^24^, as is certainly the case for our study. On average, the fraction of primary pollutants in PM_2.5_ is greater than that of secondary aerosols over the same transport distance during windy conditions. Thus, dilution is an important factor in the reduction of particulate pollution in polluted urban atmospheres.

Reddy and Venkataraman^43^ suggest that BC residence times vary as a function of precipitation, from roughly 7 to 10 days during dry conditions to about 5 days or less during wet periods. Nevertheless, our results show that equivalent BC concentrations were not significantly correlated with rainfall (Table 1). About 20% of the total sampling days were classified as rainy, so the effect of precipitation on equivalent BC concentrations is relatively unimportant. Previous studies show that wind direction is also a governing factor for BC reduction^40^,^43^. However, our study shows uncorrelated relationships between wind direction and air pollutants (i.e. BC and PM_2.5_), which indicates that our sampling site is mainly affected by local emissions rather than long-range transported emissions (Table 1)^44^. Compared to wind direction and wet precipitation, wind speed is more effective in reducing BC aerosol at the sampling site, and its concentration is highest during periods with low wind speeds.

## Conclusions

In summary, although substantial efforts have been made by the Beijing government to reduce air pollution since the 1990s, the focus has largely been on reductions of emissions from fossil and solid fuel combustion, while the contribution from secondary aerosols has been relatively neglected due to the lack of relevant long-term scientific data to explain its role in air pollution. To fill this gap in knowledge and to assist in making more clearly targeted policies, we conducted a 5-year real-time study on BC at an urban site in Beijing to identify its contribution to haze formation. We found significantly lower equivalent BC fractions in PM_2.5_ on haze days compared to those on non-haze days from 2010 to 2014, which directly confirms that primary aerosols (i.e. BC) have a relatively smaller contribution to haze formation and proves that secondary aerosols have a significantly greater contribution to Beijing air pollution. Higher rates of coal combustion in Beijing were reflected in the inter-seasonal trends of averaged BC concentration, which show BC levels increased dramatically in the cold seasons compared to in warm seasons. In contrast, the slight decrease in inter-annual BC emissions was mainly attributable to the shift in fuel source (from coal stoves to petroleum gas stoves), the expanded use of centralized heating system, as well as the implementation of new emission regulations for gasoline and diesel vehicles over the past 5 years. Such policies will eventually lead to a decrease in BC emissions in urban Beijing in the near future, but are not expected to result in a large reduction in the formation of haze in Beijing. Wind is more efficient in dispersing PM_2.5_ than BC, which may explain why secondary aerosols are not formed to the same degree during windy conditions as during stable conditions when compared over the same transport distance^20^. The analysis in this study is valuable due to its general nature, as it was conducted with a 5-year online datasheet. Also, it may aid the government in generating targeted mitigation policies to reduce haze pollution in Beijing.

## Materials and Methods

### Sampling site and air pollution measurement

The equivalent BC measurement was conducted from January 1, 2010 to December 30, 2014 at the Beijing Academy of Science and Technology (S_1_, N39° 56′50.11″, E116° 18′8.82″), which is located beside the west 3^rd^ highways in Beijing^24^,^31^,^45^,^46^. The station is 50 m away from the west 3^rd^ highways, and the sampling equipment was set up on an office building roof 30 m aboveground. The PM_2.5_ measurements were obtained at the Wanliu monitor station (S_2,_ N39° 57′31.97″, E116° 18′1.29″), which is 1.4 km north of the S_1_ station and 500 meters away from the west 3^rd^ highways (Figure S1).

Equivalent BC mass concentration was continuously measured using an Aethalometer-31 (Magee Scientific, USA) with a PM_2.5_ impactor inlet, which has 7 wavelengths (370, 470, 520, 590, 660, 880 and 950 nm)^17^. The flow rate was set to 50 mL min^−1^, with a time resolution of 5 min. The equivalent BC measurements were conducted at the 880 nm wavelength, which is considered as the standard channel to determine equivalent BC mass concentration, because absorption of other aerosols (e.g. organic aerosols) is negligible at 880 nm^47^. The Aethalometer aspirates ambient air using its inlet tube and the equivalent BC concentration is estimated using the Mass Absorption Coefficient (MAC) for the conversion of light absorption coefficient into mass concentration. The MAC value is calculated as follows MAC = 14625/λ and MAC approximates 16.6 m^2^ g^−1^ that is recommended by the manufacturer at 880 nm wavelength^48^. This value has been applied in several previous equivalent BC measurements of haze in Shanghai^42^ and Beijing^26^ These data were automatically recorded in the flash card of the instrument and displayed on the screen. The calibration of the flow rate was done quarterly under normal conditions^47^,^48^.

The Aethalometer has a great advantage in that it measures equivalent BC concentrations with multi-wavelengths. However, it also has its disadvantages due to several artifacts of filter-based measurement: scattering aerosols on the filter could enhance the absorption coefficient, which would consequently increase BC concentration; and the non-scattering aerosols could cause a decrease in measured BC concentration as the filter load increases under highly polluted conditions^49^. Several previous studies have reported scattering aerosols (i.e., NH_4_^+^, SO_4_^2−^, NO_3_^2−^) would impact the AE31 measurement and results in an overestimate of BC value regardless of mixing state^50^,^51^,^52^. For example, the particle scattering corrections required for Aethalometer range from about ~1 to 4% according to Bond^50^ based on Nephelometer mid wavelength measurement, ~3 to 11% for Arnott^51^ based on a projected scattering at the specific wavelength, as well as 2–6% for Olson^52^ based on a Nephelometer mid wavelength scattering measurement. These corrections are wavelength dependent; however the lowest scattering occurs in the near infrared range. Thus, it is expected that the maximum variability for our BC data associated with a scattering correction change resulting from a PM composition is ~10% of the relative change in scattering. On the other hand, the loading artifact can be lead to a underestimate of our BC data(~10%)^49^, which has a tendency to cancel out the overestimate caused by particle scattering. In addition, the reported values are daily averages which account for multiple filter advancements results being averaged. This can result in a systemic under reporting of light absorption, However, all daily averages are subject to the this same bias thus can be compared to each other to understand their daily trends. Nevertheless, the differences between equivalent BC fractions in PM_2.5_ during haze versus non-haze days are not likely to be biased by this systemic error.

Daily PM_2.5_ mass concentrations from 2010 to 2014 were measured by an online Tapered Element Oscillating Microbalance 1405(TEOM-1405, Thermo Scientific, USA) with a Filter Dynamic Measurement System (FDMS), which is routinely calibrated by the manufacturer^53^. Particles were collected at a flow rate of 16.7 L min^−1^ on a glass-fiber filter tape with a PM_2.5_ cyclone inlet and exposed to the mass transducer for concentration measurement. The mass transducer contains a hollow, tapered ceramic element fixed at one end and a filter attached at the other end. As particles collect on the filter, the oscillation frequency decreases proportionally to the mass added to the filter^54^. The base mass concentration is calculated as the mass added to the TEOM filter (as measured by the change in frequency) divided by the volume of air sampled during the base cycle. The TEOM data were corrected for sampling losses with the Volatile Correction Module, which has been reported as the TEOM reference equivalent method and is compatible with the FDMS datasheet^55^,^56^.

### Meteorological data collection

The meteorology variables (temperature, relative humidity (RH), pressure, wind speed and wind direction) and visibility were monitored at the equivalent BC sampling site simultaneously. Daily atmospheric pressure, temperature, wind speed, wind direction and relative humidity (RH) were recorded using a Kestrel 3000 meteorological station (Kestrel Instrument, USA). Outdoor visibility was measured using a Model 6000 visibility sensor (Belfort Instrument, USA). Rainfall data was collected from Beijing Municipal Water Affairs Bureau (http:// hdsw.bjhd.gov.cn/zxfw). We examined the impact of meteorological on haze occurrence frequency during the measurement period, as recommended by standard methods in China for observing and forecasting haze weather (QX/T 113-2010). Haze was defined as a visibility of <10 km, relative humidity of <95%, and a PM_2.5_ mass concentration that over 75 μg m^−3^.

### Statistical analysis

Summary statistics for pollutant data are presented as the mean and standard error for each day. Pearson correlation was conducted to investigate the impacts of meteorological variables (temperature, RH, pressure, wind speed, and wind direction) on equivalent BC variations. Student’s t-tests were used to estimate the mean differences in equivalent BC mass concentration and equivalent BC fractions in PM_2.5_ on haze days versus non-haze days. All statistical analyses were performed using SPSS V13.0.

## Additional Information

**How to cite this article**: Liu, Q. *et al.* Temporal variations of black carbon during haze and non-haze days in Beijing. *Sci. Rep.* **6**, 33331; doi: 10.1038/srep33331 (2016).

## Supplementary Material

Supplementary Information

## Figures and Tables

**Table 1:** Pearson correlation coefficients (r) for equivalent BC concentration (μg m^−3^), PM_2.5_ mass concentration (μg m^−3^) and eBC/PM_2.5_ ratio (%) with temperature(^o^C),atmospheric pressure (hPa), relative humidity (%), rainfall (mm), wind speed (m s−1), and wind degree.

	eBC	PM_2.5_	eBC/PM_2.5_
Temperature	−0.042	−0.062	0.135
Pressure	−0.040	0.017	−0.098
Relative humidity	0.227	0.391	−0.076
Wind speed	−0.474**	−0.464**	−0.013
Wind degree	−0.235	−0.271	0.037
Rainfall	−0.022	−0.009	−0.023

^**^p < 0.001; eBC: equivalent BC.
