# Supplementary material for: Temporal variations of black carbon during haze and non-haze days in Beijing
Source: Sci Rep. 2016 Sep 16;6:33331. doi: 10.1038/srep33331 (PMC5025889; doi:10.1038/srep33331)
Supplement: Supplementary Information [file srep33331-s1.pdf]

Supplementary Information

Temporal variations of black carbon during haze and non-haze days in Beijing

Qingyang Liu[1,3], Tangming Ma[1], Michael R Olson[3,] Yanju Liu[2], Tingting Zhang[2], Yu Wu[1], James J. Schauer[3,4]

[1]College of Biology and the Environment, Nanjing Forestry University, Nanjing, China

[2]Beijing Center for Physical and Chemical Analysis, Beijing, China

[3] Environmental Chemistry and Technology Program, University of Wisconsin-Madison, Madison, WI, USA

[4]Wisconsin State Laboratory of Hygiene, University of Wisconsin-Madison, Madison, WI, USA

E-mail: liuqingyang0807@aliyun.com

Tel/Fax:：+86-25-8547 7638

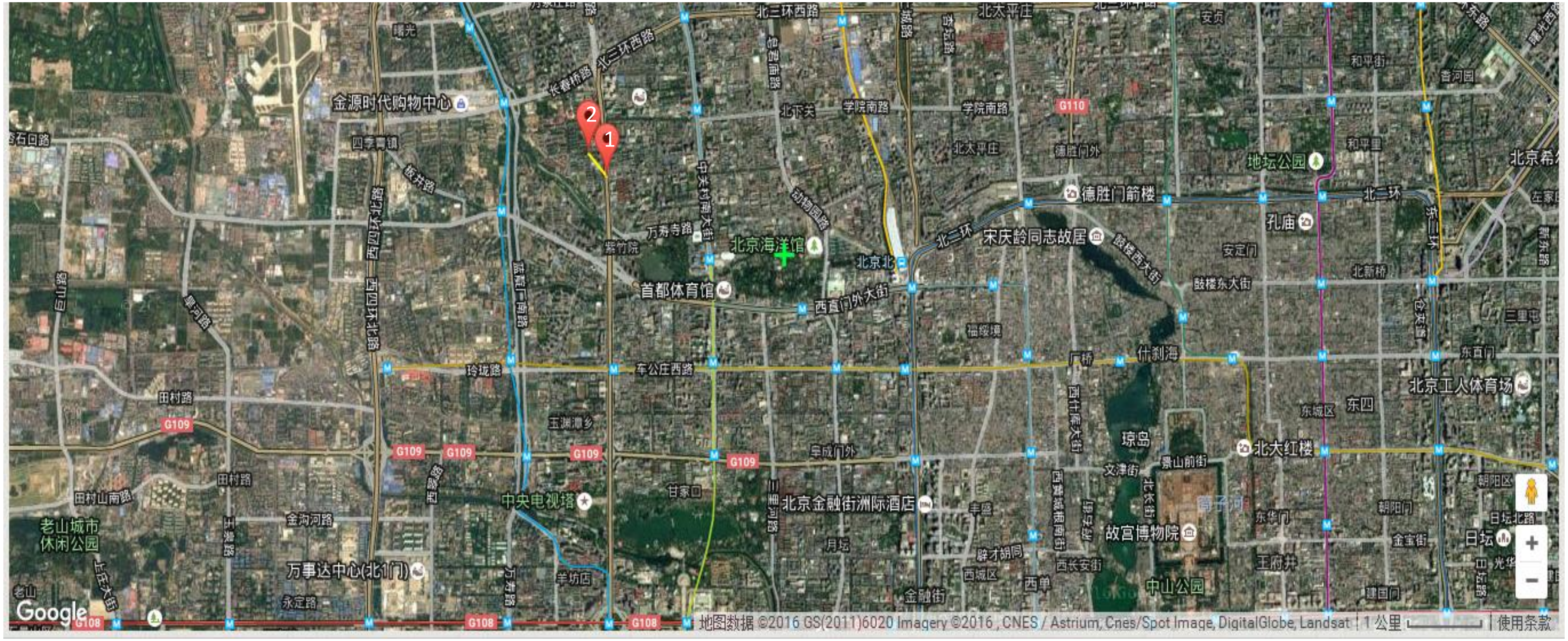

Figure S1. Sketch map of sampling sites. The sampling locations for equivalent BC(1) and $PM_{2.5}$ (2) measurements. Image was from Google Map in Chinese Edition. Map data: GS(2011). Imagery: CNES/Astrium, Cnes/Spot Image, Digital Globe, Landsat.

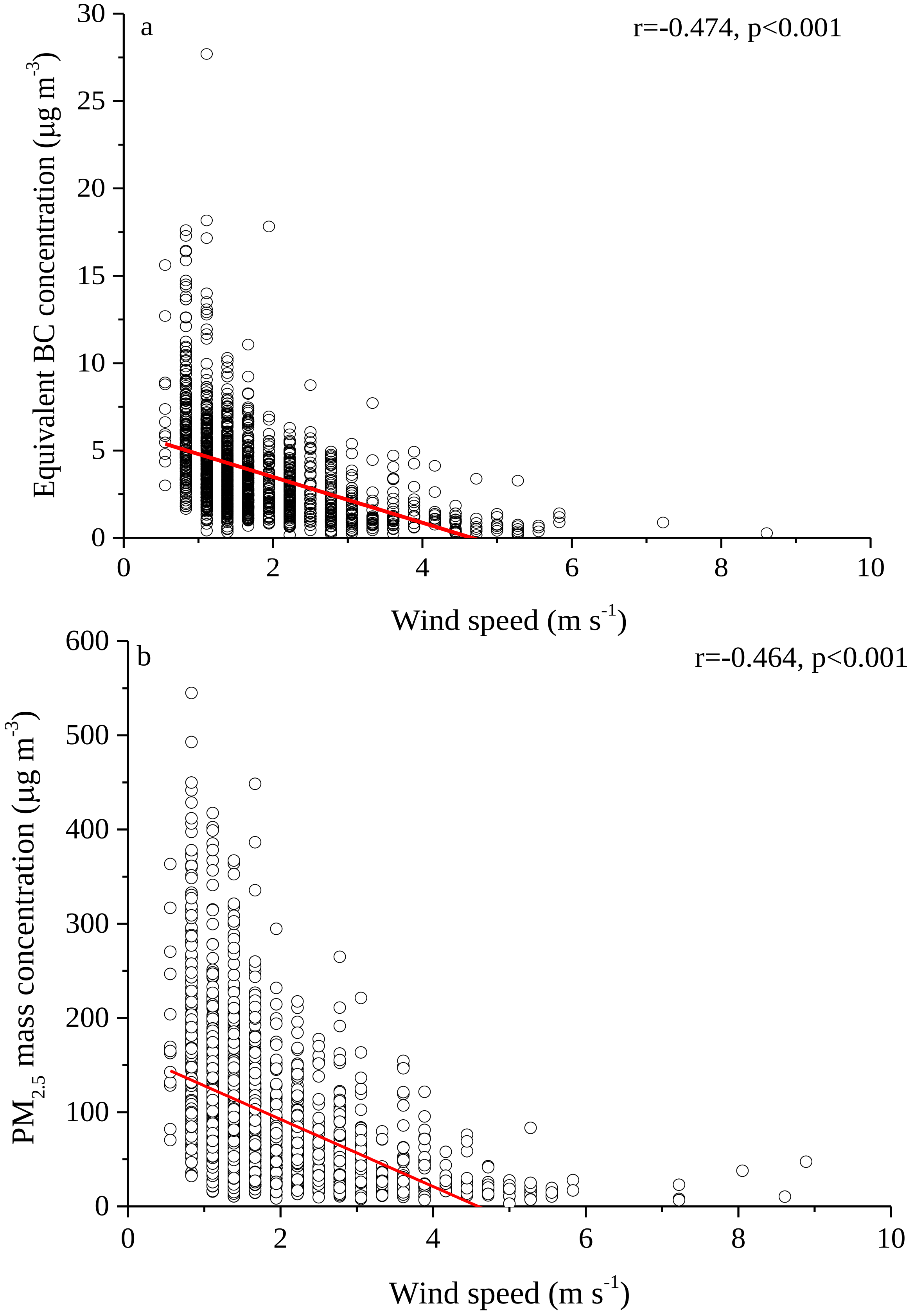

Figure S2. Relationship of equivalent BC concentration (a), $PM_{2.5}$ mass concentration (b) and wind speed at an urban site of Beijing from 2010 to 2014.

Table S1. Seasonal trends of equivalent BC concentration, $PM_{2.5}$ mass concentration and equivalent BC/$PM_{2.5}$ in Beijing from 2010 to 2014. The ranges in parentheses represent the daily average minimum and maximum values.

	Season	2010	2011	2012	2013	2014
eBC ($\mu g\ m^{-3}$)	Spring	3.71±0.25 (0.51-12.77)	3.09±0.22 (0.52-10.17)	3.50±0.19 (0.26-8.35)	2.13±0.11 (0.37-5.56)	2.47±0.15 (1.09-8.33)
	Summer	4.66±0.15 (1.96-8.52)	4.10±0.14 (1.80-7.35)	3.63±0.20 (0.33-7.53)	2.36±0.10 (0.57-5.16)	3.11±0.13 (1.02-5.57)
	Autumn	5.41±0.42 (0.61-16.44)	4.16±0.26 (0.52-9.03)	4.21±0.30 (0.37-10.13)	3.20±0.21 (0.68-8.96)	2.80±0.16 (0.87-6.10)
	Winter	5.97±0.51 (0.66-18.16)	3.74±0.37 (0.24-14.73)	3.50±0.32 (0.16-12.70)	5.74±0.74 (0.27-27.69)	3.62±0.28 (0.18-10.88)
$PM_{2.5}$ ($\mu g\ m^{-3}$)	Spring	85±5 (14-240)	64±5 (8-280)	91±6 (16-284)	91±7 (16-284)	92±6 (10-318)
	Summer	104±5 (16-243)	103±6 (11-292)	88±5 (8-237)	80±5 (14-294)	70±5 (12-218)
	Autumn	107±9 (10-374)	108±9 (13-357)	75±6 (3-280)	102±7 (15-314)	96±8 (7-387)
	Winter	108±9 (12-442)	104±10 (11-493)	102±8 (12-429)	127±13 (7-545)	145±11 (7-450)
eBC/$PM_{2.5}$ (%)	Spring	4.8±0.2 (1.5-20.3)	5.6±0.4 (1.6-23.6)	6.2±0.7 (0.2-26.7)	3.0±0.1 (1.9-5.8)	3.3±0.3 (0.7-9.6)
	Summer	6.4±0.4 (2.3-22.4)	5.1±0.4 (2.0-25.7)	5.8±0.6 (0.6-26.9)	3.4±0.1 (1.7-6.7)	5.5±0.3 (1.9-13.8)
	Autumn	6.6±0.4 (2.6-22.3)	4.9±0.3 (1.9-12.8)	5.5±0.1 (3.1-8.3)	3.7±0.1 (1.6-6.9)	5.4±0.3 (3.0-13.4)
	Winter	5.4±0.1 (1.4-9.9)	3.5±0.1 (1.7-8.7)	3.1±0.2 (0.4-14.0)	4.8±0.3 (2.1-18.1)	2.9±0.1 (1.1-5.6)

SE: standard error; eBC: equivalent BC.

Table S2. Annual data summary of equivalent BC concentration, $PM_{2.5}$ mass concentration and equivalent BC/$PM_{2.5}$ in Beijing from 2010 to 2014.

	Year	Mean ±SE	Min	5%ile	10%ile	50%ile	75%ile	98%ile	Max
	2010	4.82 ±0.18	0.51	1.06	2.60	4.25	6.05	14.43	18.16
eBC	2011	3.80 ±0.15	0.24	0.77	1.89	3.59	5.10	9.33	14.73
(μg m^{-3})	2012	3.44 ±0.13	0.16	0.43	1.64	3.22	4.71	8.25	12.70
	2013	2.95 ±0.17	0.27	0.73	1.65	2.25	3.29	11.19	27.69
	2014	3.27 ±0.14	0.18	1.01	2.06	3.11	4.13	9.46	10.88
	2010	101 ±4	10	19	46	82	132	342	442
$PM_{2.5}$	2011	95 ±4	8	16	35	71	132	315	493
(μg m^{-3})	2012	90 ±4	3	15	35	75	124	264	429
	2013	101 ±4	7	19	47	75	127	344	545
	2014	99 ±4	7	19	43	76	125	341	450
	2010	5.7 ±0.2	1.4	3.1	4.2	5.0	6.4	13.4	22.4
eBC/$PM_{2.5}$[c]	2011	4.7 ±0.1	1.6	2.1	3.1	4.0	5.6	11.0	25.7
(%)	2012	5.0 ±0.2	0.2	1.0	2.2	3.9	6.0	20.3	26.9
	2013	3.6 ±0.1	1.6	2.1	2.6	3.3	4.1	6.9	18.1
	2014	4.3 ±0.2	1.1	1.8	2.7	3.8	5.0	10.6	13.8

SE: standard error; eBC: equivalent BC.

Table S3. Annual vehicle count in Beijing. The data were obtained from the Beijing Municipal Bureau of Statistical website (http://www.bjstats.gov.cn/).

Year	On-road gasoline numbers (10^4 units)	On-road diesel numbers (10^4 units)
2010	452.9	28
2011	473.2	25.1
2012	495.7	24.3
2013	518.9	24.8
2014	532.4	26.7

.

Table S4. Data summary of equivalent BC concentration and equivalent BC/$PM_{2.5}$ in Beijing on haze and non-haze days from 2010 to 2014.

Component	Year	Haze		Non-haze		Significance
		n	Mean ±SE	n	Mean ±SE	
	2010	153	6.63 ±3.35	146	2.88 ±1.74	p<0.001
eBC (μg m^{-3})	2011	129	5.44 ±2.13	113	1.99 ±1.11	p<0.001
	2012	144	4.35 ±2.32	133	2.61 ±1.77	p<0.001
	2013	122	4.72 ±1.49	147	1.70 ±0.73	p<0.001
	2014	124	3.85 ±1.95	102	2.20 ±0.96	p<0.001
	2010	153	4.5 ±1.1	146	6.9 ±3.6	p<0.001
eBC/$PM_{2.5}$(%)	2011	129	3.6 ±1.3	113	5.8 ±3.4	p<0.001
	2012	144	3.8 ±1.8	133	7.1 ±5.8	p<0.001
	2013	122	2.8 ±0.7	147	3.9 ±1.2	p<0.001
	2014	124	2.7 ±1.1	102	5.7 ±2.5	p<0.001

SE: standard error; Haze was defined as a visibility of <10 km, relative humidity of <95%, and a $PM_{2.5}$ mass concentration over 75 μg m^{-3}; eBC: equivalent BC.

Table S5. Annual mean values and standard deviation (SD) of temperature, wind speed, and rainfall (the number of rainy days) from 2010 to 2014. The ranges in parentheses represent the daily average minimum and maximum values.

Year	2010	2011	2012	2013	2014
Temperature (℃)	11±12	12±11	12±11	12±11	15±10
	(-14-32)	(-8-33)	(-12-35)	(-10-35)	(-7-32)
Relative Humidity (%)	56±19	56±22	56±21	53±20	52±19
	(16-92)	(12-96)	(12-98)	(10-94)	(8-93)
Pressure (hPa)	1023±9	1023±11	1022±10	1022±10	1015±9
	(1002-1046)	(1003-1048)	(1001-1045)	(1000-1050)	(996-1037)
Wind speed (m s^{-1})	1.8±0.9	1.8±1.0	1.7±0.9	1.6±0.8	2.4±1.1
	(0.6-5.8)	(0.6-5.8)	(0.6-5.8)	(0.6-5.6)	(0.6-8.9)
Rainfall (mm)	569	658	617	498	484
